# Straw strip mulching boosts potato yields by enhancing soil moisture and water use efficiency

**DOI:** 10.3389/fpls.2025.1659236

**Published:** 2025-10-28

**Authors:** Qian Chen, Lei Chang, Fanxiang Han, Khuram Shehzad Khan, Yuwei Chai, Shouxi Chai, Linlin Wang, Jiantao Ma

**Affiliations:** ^1^ Gansu Provincial Key Laboratory of Aridland Crop Science, Lanzhou, China; ^2^ College of Agronomy, Gansu Agricultural University, Lanzhou, China; ^3^ College of Geography and Environmental Engineering, Lanzhou City University, Lanzhou, China

**Keywords:** straw strip mulching, plastic film mulching, water consumption characteristics, yield, potato

## Abstract

**Introduction:**

Water scarcity is a critical constraint limiting potato production in semi-arid rainfed areas. Mulching practices are recognized as effective water conservation strategies; Here, we investigated the effects of mulching practices on soil moisture and their impact on potato yield.

**Methods:**

A two-year field experiment (2018 – 2019) was conducted with five treatments, traditional bare-land planting without mulching (CK); large ridges and small furrows with only ridges mulched with black plastic film in fall (FPM); a partial-field mulching using whole cornstalks in straw mulching strips that alternate with planting strips without mulch in fall (FSM); large ridges and small furrows with only ridges mulched with black plastic film in spring (SPM); a partial-field mulching using whole cornstalks in straw mulching strips that alternate with planting strips without mulch in spring (SSM). We measured soil water storage (0–200 cm), yield, water-use efficiency (WUE), and stage-specific water consumption (WC).

**Results:**

Study results demonstrated that SSM and SPM significantly increased soil water storage (0–200 cm) by 6.7% and 8.4%, yield by 14.7% and 25.1%, and water-use efficiency (WUE) by 9.2% and 14.3%, respectively, compared to CK. Compared to spring mulching, the fall mulching outperformed in improving soil water retention and yield, increasing soil water storage by an average of 10.2% vs. 4.9%, and fresh potato yield by 17.8% vs. 11.8%. SSM and SPM reduced water consumption (WC) during the early growth stage (planting-to-budding) by 8.2–9.8%, conserving water for later use. This conserved water was then available during the critical yield-forming period, leading to increased WC during budding-to-tuber expansion by 10.3–11.3%. SSM increased WC more than FSM (12.3% vs. 10.2%), while FPM increased WC more than SPM (20.3% vs. 13.1%).

**Discussion:**

The findings indicated that both the straw strip mulching (SM) and plastic film mulching (PM) optimized the water consumption structure. Fall mulching generally outperformed spring mulching because it captured and conserved autumn and winter precipitation more effectively, resulting in higher soil water storage at planting. Both straw and plastic film mulching improve water use and potato yields, with fall application were most effective. However, for sustainable production, straw strip mulching is recommended, as it offers both high crop yields and significant environmental benefits.

## 1 Introduction

In the arid and semi-arid areas of China, dryland agricultural production faces significant challenges due to scarce and unpredictable precipitation, high evaporation rates, and low rainwater utilization efficiency, leading to low and unstable farmland productivity (Gan et al., 2013; Peng et al., 2020; Yang et al., 2023). Potato (*Solanum tuberosum* L.), a major economically important crop in the semi-arid areas of Northwest China, plays a critical role in ensuring national food security (Li et al., 2022a; Zou et al., 2020). This region contributes approximately 36% of China’s total potato production, highlighting its importance in the agricultural sector (Hou and Li, 2019; Hou et al., 2020; Qin et al., 2022). However, persistent drought conditions and soil water deficits severely limit the achievement of and a stable potato yield as well as the sustainable development of the potato industry (Li et al., 2021). To address these challenges, it is imperative to develop efficient mulching cultivation techniques and water-saving measures that can effectively reduce soil water evaporation, optimize soil water allocation, and enhance water use efficiency (WUE). Implementing such strategies is essential for improving potato yields and ensuring sustainable agricultural production in this water-scarce region.

Mulching has been widely recognized for its ability to promote plant growth and development, enhance drought tolerance, improve crop yield, and increase WUE (Billman and Campbell, 2023; Fang et al., 2021; Qin et al., 2015; Verhulst et al., 2011; Wang et al., 2018; Zhang et al., 2022). Plastic film mulching and straw mulching have become the main cultivation techniques in Northwest China due to their effectiveness in conserving soil moisture and enhancing soil water storage (Sabet et al., 2022; Singh et al., 2015). Numerous studies have demonstrated the significant benefits of plastic film mulching, including reducing soil water evaporation by 14.5%–29.8% (Gu et al., 2017; Li et al., 2022b; Yang et al., 2023), increasing soil water storage capacity by 13-21% (Zhang et al., 2020a), and optimizing soil water content across different crop growth stages. These improvements have led to substantial increases in potato yield (19.7%–51.6%) and WUE (16.4%–111.7%) (Obour et al., 2022; Xiong et al., 2020; Zhou et al., 2011). Despite these agronomic benefits, long-term reliance on plastic film mulching has been linked to adverse environmental impacts. For instance, residual plastic film in the soil is difficult to reclaim, which can hinder water and nutrient absorption, reduce crop transpiration, and lower the efficiency of soil moisture utilization (Chen et al., 2015; Zhao et al., 2022). Meta-analyses by Zhang et al. (2020a) revealed that prolonged plastic film use decreased soil moisture evapotranspiration capacity by 2%, reduced soil moisture infiltration by 8%, and lowered soil organic matter by 0.8%. In some polluted areas, cotton yields were estimated to decline by 6%-10%, highlighting the adverse impacts on crop productivity and the sustainable development of agriculture (Chen et al., 2015; Zhao et al., 2022). Additionally, for potato cultivation, the high temperature and humidity conditions under black plastic film mulching (PM) can exacerbate the occurrence of late blight, negatively affect tuber expansion and ultimately reduce potato yields. These limitations highlight the urgent need for alternative mulching practices that sustain productivity while minimizing environmental risks.

Straw mulching has been widely adopted in potato production across the semi-arid regions of Northern China due to its effectiveness in optimizing water utilization throughout all growth stages (Dong et al., 2018). By covering the soil surface, straw mulch reduces direct evaporation, promotes greater rainfall infiltration, and enhances soil water storage, thereby ensuring more stable water availability during critical growth stages. Previous studies have shown that straw mulching can enhance maize (*Zea mays* L.) and wheat (*Triticum aestivum* L.) yield and WUE (Feng et al., 2019; Li et al., 2013, 2019; Zhang et al., 2020b). The water-saving effects arise primarily from suppressed soil evaporation, which shifts water use toward crop transpiration, thereby improving water-use efficiency. Additionally, it moderates soil temperature by blocking excessive solar radiation (Ma et al., 2022), creating a more favorable environment for potato tuber formation and expansion. As a result, straw mulching has been reported to increase potato yields by 4.5%–34.0% and WUE by 6.8%–21.5% (Hou and Li, 2019; Shafiq and Kaur, 2021). These advantages make straw mulching a sustainable and effective practice for boosting potato productivity in water-scarce regions.

Northwest China is rich in maize straw resources, but most of them are not fully used. Traditional full straw mulching is regarded as one of the best ways to conserve soil moisture in Northern China by increasing the rainwater infiltration capacity and improving the utilization efficiency of corn straw (Qin et al., 2021). However, traditional straw mulching practices have been shown to not only delay crop by reducing soil temperature growth (Zhao et al., 2019b), particularly in cooler climates, but also to increase mechanical difficulties and farming costs. This highlights the need to develop innovative straw mulching techniques that balance the dual objectives of conserving soil moisture and mitigating the negative impact of reduced soil temperature. Additionally, these new methods must be compatible with mechanized tillage systems to ensure practicality and scalability in the semi-arid areas of Northern China (Gao et al., 2022a).

To address these challenges, the straw strip mulching system (SM) technique is introduced as an alternative to straw-mulched strips with bare planting strips. This innovative approach has demonstrated several advantages: it significantly reduces total water consumption during the potato reproductive period by 6.1%-13.2%, increases potato yield by 10.5%–34.2%, and enhances WUE by 8.9%–29.8% (Chen et al., 2019a). In addition, SM promotes nutrient return through straw decomposition, improves soil fertility, and enhances soil water storage (Chang et al., 2020; Song et al., 2024). While straw degradation can initially increase nitrogen demand and lead to temporary competition with the crop, this effect is mitigated over time as decomposed straw contributes to soil organic matter and nutrient release, maintaining nutrient balance in the longer term (Ninkuu et al., 2025). Previous studies have also shown that straw mulching enhances soil enzyme activity (Song et al., 2024) and improves soil structure in potato farmlands (Ma et al., 2024), creating favorable conditions for potato growth and development, as a result, and thus increases potato yield. The straw strip mulching system is considered a promising and sustainable approach for enhancing agroecosystem productivity in the arid and semi-arid agricultural areas of Northwest China (Chai et al., 2022b).

Although both plastic film and straw strip mulching have been studied, limited knowledge exists on how straw strip mulching (SM) regulates water consumption dynamics across different potato growth stages in semi-arid environments. Most existing research has focused on either full plastic film or whole-field straw mulching, with few studies systematically comparing the stage-specific soil water partitioning and water-saving mechanisms between straw strip mulching systems and conventional plastic film mulching (PM). Moreover, the optimal timing for mulching application (fall vs. spring) remains unexplored for potato production under water-limited conditions. To address this gap, we conducted a two-year field experiment (2018–2019) in the semi-arid area of Northwest China to investigate the effects of various mulching practices and stages on potato water consumption and yield. The study compared SM applied in fall and spring, large ridges and small furrows, with only ridges covered by PM in fall and spring, and traditional bare-land planting without mulching. The objectives were to: (a) quantify the effects of SM on soil water partitioning during key potato growth phases; (b) compare water-saving mechanisms between SM and PM systems; and (c) determine the optimal mulching timing for maximizing water-use efficiency and yield. This research aims to provide valuable insights into optimizing mulching techniques to enhance water conservation, crop productivity, and agricultural sustainability in water-scarce environments.

## 2 Materials and methods

### 2.1 Experimental site description

**Figure 1:**
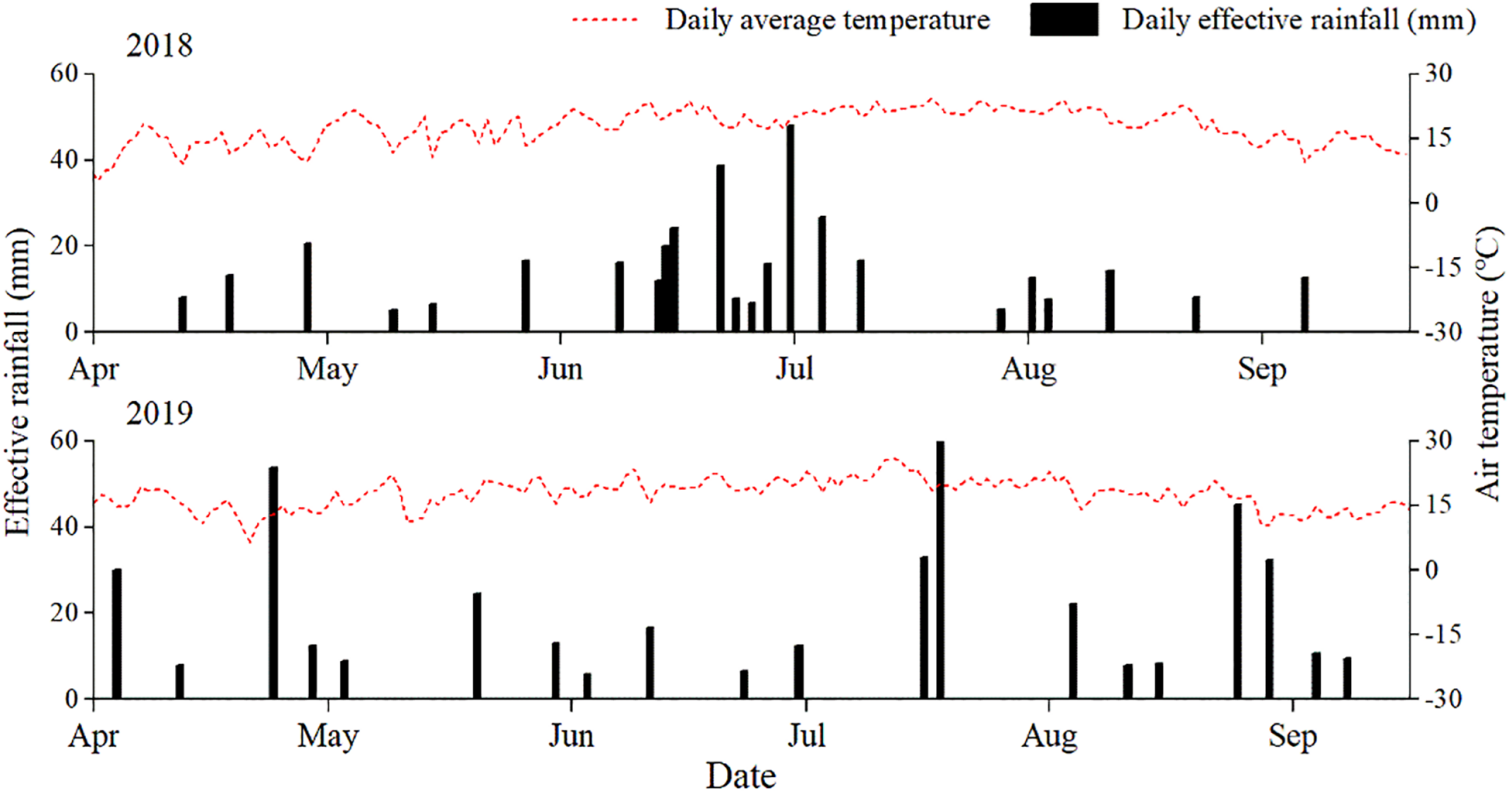
Daily average temperature and daily effective rainfall during the potato growing season in experimental site during 2018–2019.

The field experiment was conducted in 2018 and 2019 at the Tongwei Modern Dryland Circular Farming Experiment Station in Dingxi City, Gansu Province, China (35°11′ N, 105°19′ E). The study area is characterized by a typical arid inland climate of Northwest China, where crops ripen once a year. With an average annual temperature of 7.2 °C and altitude of 1740m. The region receives an annual sunshine duration ranging between 2100 and 2340h, with a frost-free period lasting between 120 and 170d. The average annual rainfall is 390.7mm, with over 60% of the rainfall occurring between July and September. The amount of precipitation and atmospheric temperature in the area during the test are shown in **Figure 1**. The average atmospheric temperatures were 16.0 °C and 16.0 °C. The precipitation during the potato growth period (from planting to harvest) was 442mm in 2018 and 439mm in 2019, while the effective rainfall (≥5 mm) were 365mm and 424mm, respectively (**Figure 1**). Effective rainfall accounted for 95.8% and 83.1%of the total rainfall during the potato growth periods in 2018 and 2019, respectively.

The annual mean soil evaporation is 1500mm, making the region highly susceptible to spring droughts. According to the USDA texture classification system, the soil at the experimental site is classified as loess soil (Corral-Pazos-de-Provens, 2022). The average bulk density of 0–20 cm soil was 1.25g cm^−3^. The physical and chemical properties of the soil in the experimental area are detailed in **Table 1**. The dryness conditions during potato growth periods were characterized by analyzing both rainfall amounts and derived drought indices (DI) across experimental years. DI > 0.35 as a wet year, DI < −0.35 as a drought year, and −0.35 ≤ DI ≤ 0.35 as a normal year, in which the DI in 2018 and 2019 were 1.1 and 1.6, respectively, as a result, 2018 and 2019 were categorized as wet years.

**Table 1:** Basic physical and chemical properties of the soil in experimental site.

Index	Bulk density g·cm^-3^	Organic matter g·kg^-1^	Total nitrogen g·kg^-1^	Available phosphorus mg·kg^-1^	Available potassium mg·kg^-1^	pH
Physicochemical properties	1.3	11.7	0.8	11.6	122.7	8.5

### 2.2 Experimental design and field management

**Figure 2:**
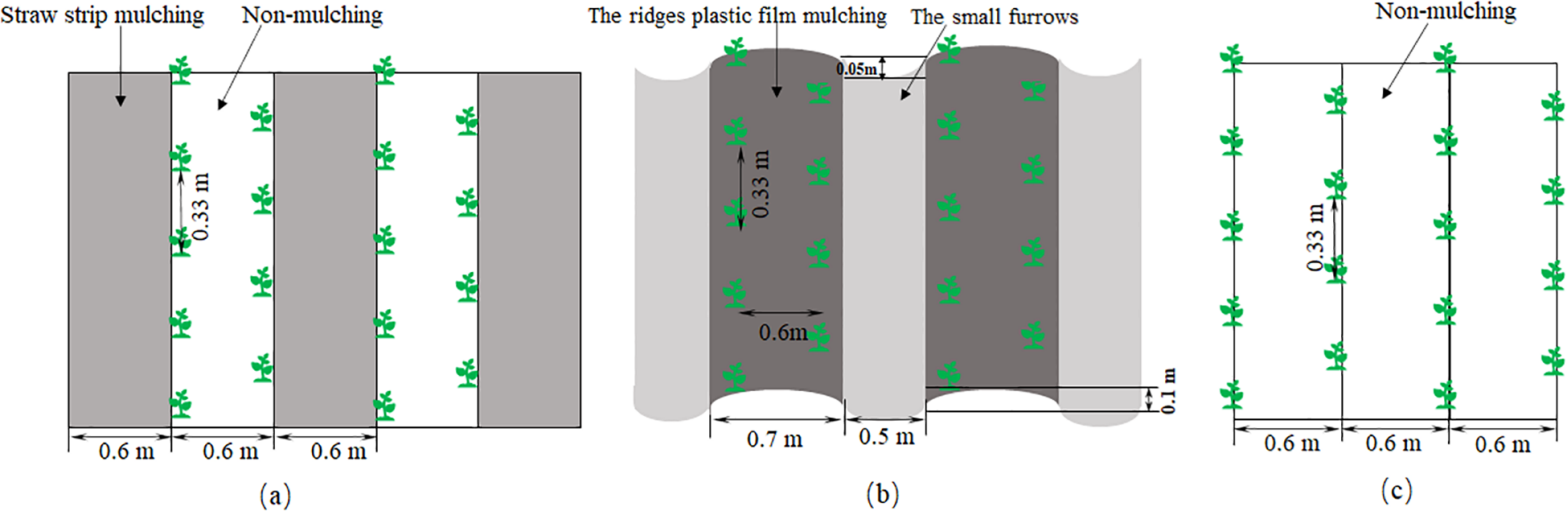
Schematic diagram of potato cultivation. **(A)** Alternating strip mulching with maize straw and bare plots with no mulching. **(B)** Alternating large ridges with black plastic film mulching and small furrows. **(C)** Traditional bare-land planting without mulching.

In this experiment, a completely randomized block design with three replicates was employed, comprising five treatments. A total of 15 plots were established in both 2018 and 2019, with each plot measuring 18m length and 6m width. The specific treatments were as follows: (1) FSM (straw strip mulching in fall): a partial-field mulching using whole cornstalks in straw mulching strips that alternate with planting strips without mulch. Alternating straw mulch strips (0.6m width) and bare plots without mulching (0.6m width) in the fall (**Figure 2A**). In mid-October 2017, following the harvest of the previous crop and subsequent rototilling and harrowing of the field, a straw mulch composed of whole corn stalks (5–10 cm thick) was applied. Each planting strip was sown with two rows of potatoes, with 33cm plant spacing and 60cm row spacing; (2) SSM (straw strip mulching in spring): Similar to FSM, but straw mulch strips were applied in the spring using whole corn stalks. The planting method was identical to that of FSM; (3) FPM (large ridges and small furrows with only ridges mulched with black plastic film in fall): Alternating large ridges (0.7m width, 0.1m height) and small furrows (0.5m width, 0.05m depth) in the fall (**Figure 2B**), only the ridges were mulched with black plastic film (1.2m width, 0.01mm thickness (Lanzhou Xinyinhuan Rubber and Plastic Products Co., Ltd, China). Two rows per ridge, row spacing 60cm. The FPM treatment followed an identical procedure in both years: rotary tillage was conducted after the harvest of the respective previous crop (i.e., 2017 for the 2018 season, and 2018 for the 2019 season), followed by mulching in mid-October. (4) SPM (large ridges and small furrows with only ridges mulched with black plastic film in spring): Similar to FPM, The planting method was identical to that in FPM (5) CK: Traditional bare-land planting with no mulching. The row spacing was 60cm, consistent with the other treatments (**Figure 2C**). In this experiment, the ground film mulch adopts the design of 0.7m for the width of the ridge and 0.5m for the width of the furrow, which is not only in line with the local conventional farming pattern, but also can effectively promote rainwater enrichment and match with the mainstream agricultural machinery. The straw strip cover adopts the equal width configuration of 0.6m for the planting belt and the cover belt, which is designed to meet the requirements of mechanized operation and ensure crop ventilation and light transmission. The choice of these dimensions is based on actual local production conditions. This experimental design allowed for a comprehensive comparison of the effects of different mulching stages (fall vs. spring) and methods (straw vs. plastic film) on soil water conservation, potato growth, and yield in the semi-arid region of Northwest China.

For the SM treatments, whole maize straw was collected from harvested fields, air-dried, and uniformly applied at a rate of 9,000 kg·ha^-1^ (equivalent to approximately 52,500 plants·ha^-1^) by hand onto the mulching strips in each experimental year. The straw was stabilized against winter winds by soil compaction at 2m intervals. Before planting in each season, systematic field preparation was carried out: the field was first cleared of weeds, followed by the application of a basal fertilizer (120kg N ha^-1^ + 90kg P_2_O_5_ ha^-1^) according to conventional local fertilization standards. After fertilization, the fertilizer was evenly mixed into the 0–30 cm layer of soil by tillage. The amount of fertilizer applied and the method of application (one-time basal application before planting) used in this experiment were in line with the general practice of dry farming in the region. The potato cultivar “Longshu No. 7” (the medium–late maturing variety) was planted at a density of 52,500 plants·ha^-1^, with an interplant spacing of 33cm on 12 April 2018 and 16 April 2019. All plants were harvested in October, and after harvesting, any plastic film residue was completely removed by hand from plots mulched with plastic film. The plots for each treatment remained unchanged throughout the two-year trial. To maintain consistency, the maize straw used in the first year was reused in the second year without adding new maize straw, ensuring an equal amount of straw mulch across both years for all straw mulching treatments.

### 2.3 Plant materials and agronomic practices

We confirm that the use of plant materials in this study complies with all relevant institutional, national, and international guidelines and legislation. The potato cultivar used in this research, ‘Longshu No. 7’ is widely cultivated in the first cropping area of Northwest China, including the eastern part of Qinghai Province, the central and eastern regions of Gansu Province, the central and southern areas of Ningxia Hui Autonomous Region, and the eastern, central, and western regions of Guangdong Province. All seed tubers were sourced from the Potato Research Institute of Gansu Academy of Agricultural Sciences, ensuring the quality and authenticity of the plant materials used in this study.

### 2.4 Measurements and methods

#### 2.4.1 Determination of soil water storage

The first measurement of soil water content (SWC, %) was conducted one day before planting. Subsequent measurements were taken at five key growth stages of potato: seedling stage (day 35 after planting), budding stage (day 60 after planting), tuber expansion stage (day 100 after planting), starch accumulation stage (day 130 after planting), and maturity stage (day 165 after planting) (Ma et al., 2024). SWC was measured in eight soil layers (0–20, 20–40, 40–60, 60–90, 90–120, 120–150, 150–180, and 180–200 cm). Deep sampling employed a 5-centimeter-diameter auger equipped with a full set of interlocking extension rods, enabling us to reach the target depth of 200cm. If rainfall occurred on the scheduled sampling day, sampling was delayed for 2–3 days to avoid interference. At each growth stage, three soil points were randomly selected for sampling in each plot in each growth stage of potato, and a new borehole was excavated at each point for each sampling event. Each sampling point was located in the middle of two potato plants. Plastic Film Mulching sampling focused on taking three random soil samples between two potato plants using a 5cm diameter handheld soil auger. For plastic film mulching treatments, three soil samples were randomly taken between two plants. For straw strip mulching treatments, three soil samples were collected from the planting strip and three from the mulching strip. Specifically, three samples were taken between two plants in the planting strip, and three samples were taken from the middle of the mulching strip beneath the straw. The SWC for straw strip mulching was calculated as the weighted average of the values from the mulching strip and the planting strip. Soil samples (M_1_) were oven-dried at 105 °C for 48 hours until a constant weight (M_2_) was achieved. The average SWC for the 0–2 m soil depth was calculated as the weighted average of the eight soil layers (Zhang et al., 2017).

The SWC (%) was determined using Equation 1:


(1) $$\text{SWC}=({\text{M}}_{1}-{\text{M}}_{2})/{\text{M}}_{2}\times 100\%$$

The soil water storage (W, mm) was determined using Equation 2:


(2) W=SWC×ρ×h×10

where *ρ* is the soil bulk density (g·cm^-3^), the average soil bulk density of each soil layer from 0–200 cm in this experiment was 1.25g cm^-3^, and *h* (mm) is the soil depth.

#### 2.4.2 Determination of water consumption

The field experiments were conducted under rainfed conditions without irrigation during the two growing seasons in 2018 and 2019. In the experimental plots, the water table was approximately 50m below the surface, making upward water flow into the root zone negligible. Given the arid and flat terrain of the study area, where local rainfall is generally low, surface runoff and seepage drainage were also considered insignificant. As a result, drainage, surface runoff, and seepage below the 200cm soil layer were assumed to be negligible in both the straw strip mulching and plastic film mulching treatments. Nearly all rainfall was retained within the 0–200 cm profile, so soil water balance was primarily governed by precipitation, evaporation, and crop water uptake. The soil water balance equation was used to calculate soil water consumption during the entire growth period, as well as the water consumption and daily water consumption intensity (WCI) in each growing stage (Wei et al., 2018).

Soil water consumption, also known as evapotranspiration. The soil water consumption was determined by the equation of soil water balance (Chen et al., 2019b; Li et al., 2004) as shown in Equation 3:


(3) WC=ΔW+I+P+Q−F

WC is the soil water consumption of the potato during the growth stages of potato, where P (mm) is the effective precipitation during the growth stages of potato, and ΔW (mm) is the change in W (mm) during the growth stages of potato. I is the amount of irrigation during the growth stages of potato (mm); Q is the amount of soil water infiltration in the tillage layer or the amount of groundwater recharge to the tillage layer in time period t (mm), when the depth of the groundwater is greater than 2.5m, the value of Q can be disregarded; F is the amount of surface runoff in time period t (mm). In this experiment, there was no irrigation, no runoff, and the groundwater was greater than 2.5m, so I, Q, and F were all zero. Soil water consumption (WC, mm) was calculated using the simplified Equation 4:


(4) WC=ΔW+P

#### 2.4.3 Determination of water consumption intensity

The WCI (mm·d^-1^), also known as daily water consumption, was determined using Equation 5 (Feng et al., 2019):


(5) WCI=WC/T

where T (d) is the number of days covered by the growth stages of potato.

#### 2.4.4 Determination of WUE

The WUE (kg ha^-1^·mm^-1^) was determined using Equation 6 (Wei et al., 2018):


(6) WUE=DY/WC

where DY (kg·ha^-1^) is the dry potato yield.

#### 2.4.5 Determination of tuber yield

Before potato harvest, 15 plants were randomly selected from each plot to evaluate key yield and quality parameters, including the number of tubers per plant, weight of individual potatoes, commercialization rate (CR) and tuber moisture content. Based on the International Commercial Potato Quality Classification Index (Chen et al., 2019a), potatoes were categorized into three grades: grade 1, large potatoes (>150g); grade 2, medium potatoes (75–150 g); grade 3, small potatoes (< 75g). Fresh tubers were dried in an oven (105 ± 0.5 °C), and the water content of tubers was calculated. The number of potatoes of each grade was counted and weighed to calculate the commercialization potato rate (CR). which was determined using the formula:


CR(%)=≥75 g of tuber weight/the total output of tuber×100%

### 2.5 Statistical analysis

W, WC, and daily WCI during each growing stage, tuber yield, and WUE data were processed using the IBM SPSS Statistics software package (Ver. 26.0, IBM SPSS Inc., Chicago, IL, USA). The assumption of normality (Shapiro-Wilk test) and the assumption of chi-square (Levene’s test) were verified (*P > 0.05*) for all datasets prior to ANOVA. A one-way ANOVA was conducted to assess the differences among treatments. After confirming the significance of ANOVA, Duncan’s multiple range test (DMRT) was used at *P ≤ 0.05*. Following statistical analysis, Microsoft Excel (Ver. 2019, Microsoft, USA) was used for data organization and preliminary calculations. Graphical representations of the results were created using Origin (Ver. 9.6, Origin Lab, Northampton, MA, USA).

### 2.6 Economic analysis

All economic values are presented in US Dollars (USD) per hectare. Conversions were made from original values in Chinese Yuan (RMB) per mu using the average annual exchange rate for 2025 (1 USD=7.1 RMB) and the standard unit conversion (1 hectare = 15 mu).

#### 2.6.1 Plant guidelines

All the plant experiments were performed according to relevant institutional, national, and international guidelines and legislations.

## 3 Results

### 3.1 Soil water storage in the 0–200 cm soil layer

**Figure 3:**
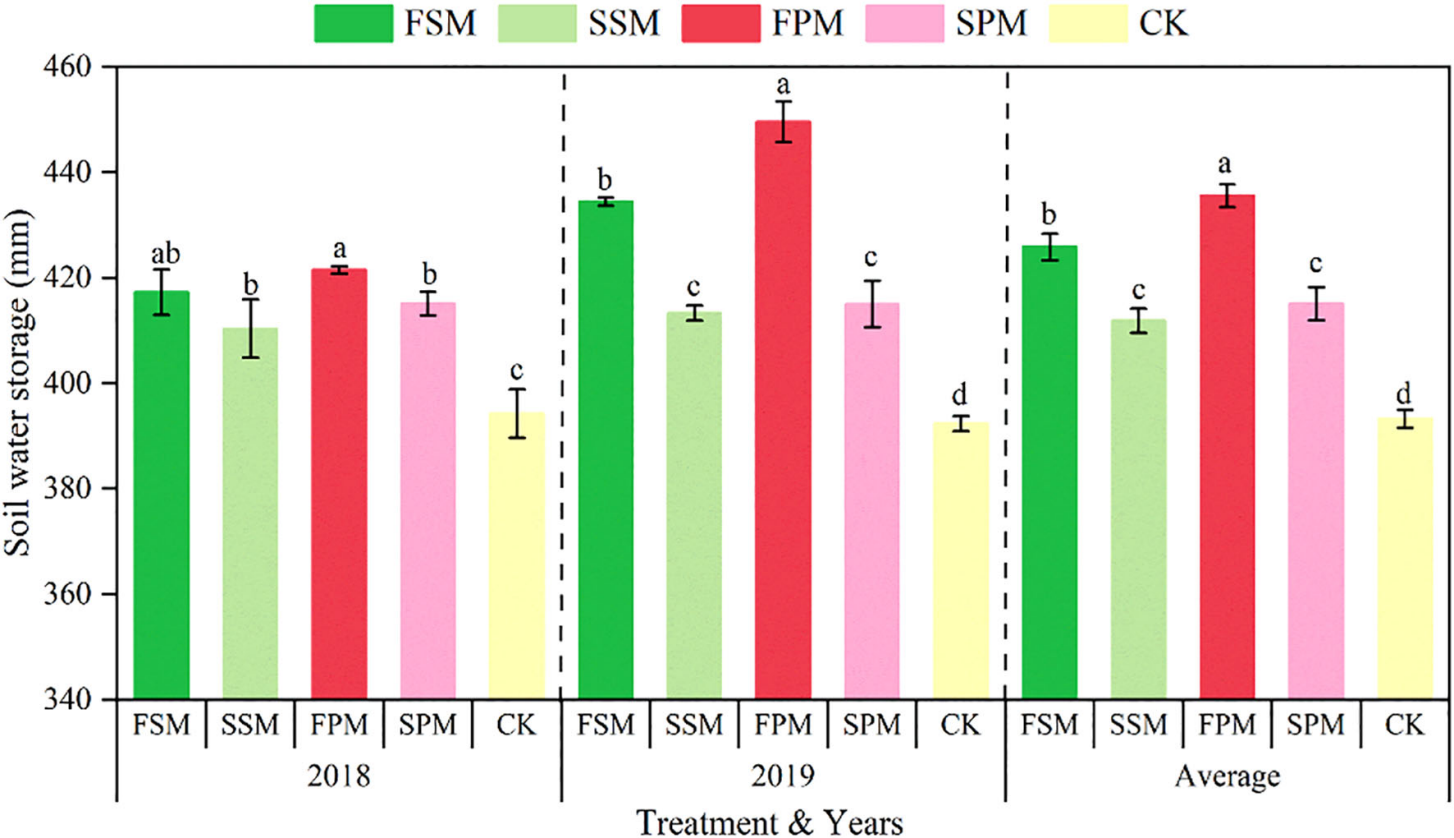
Soil water storage capacity of 0–200 cm soil during the entire potato growth stage in 2018,2019 and averaged over both growing seasons. FSM, maize straw strip mulching in fall; SSM, maize straw strip mulching in spring; FPM, large ridges with black plastic film mulching and small furrows in fall; SPM, large ridges with black plastic film mulching and small furrows in spring; CK, traditional bare-land planting without mulching. Identical lowercase letters indicate no significant differences (*P > 0.05*, duncan’s multiple range test) among potato treatments in a given year. Bars represent the standard error.

The mulching treatments significantly (*P < 0.05*) increased the W in the 0–200 cm soil layer throughout the entire growth stage (**Figure 3**). In the 2018 growing season, compared to the CK, the SM and PM treatments significantly (*P < 0.05*) increased the W by 5.3% and 6.6%, respectively. Among the different mulching stages, the increase in W followed the order: FSM (6.2%) > SSM (4.4%) and FPM (7.3%) > SPM (5.8%). Similarly, in the 2019 growing season, SM and PM significantly (*P < 0.05*) increased W by 8.1% and 10.2% compared to CK, respectively. Compared with CK, the increase in W during the different mulching stages was as follows: FSM (10.8%) > SSM (4.3%) and FPM (15.3%) > SPM (5.0%).

When averaged across the two growing seasons, the W under FSM, SSM, FPM, and SPM treatments was significantly (*P < 0.05*) higher than CK, with increases of 9.0%, 4.3%, 11.3%, and 5.4%, respectively. Overall, SM and PM increased W by 6.7% and 8.4%, respectively, while fall mulching and spring mulching increased W by 10.2% and 4.9%, respectively. In summary, fall mulching demonstrated a greater effect on W compared to spring mulching, and PM outperformed SM in both the 2018 and 2019 growing seasons.

### 3.2 Water consumption at different potato growth stages

**Figure 4:**
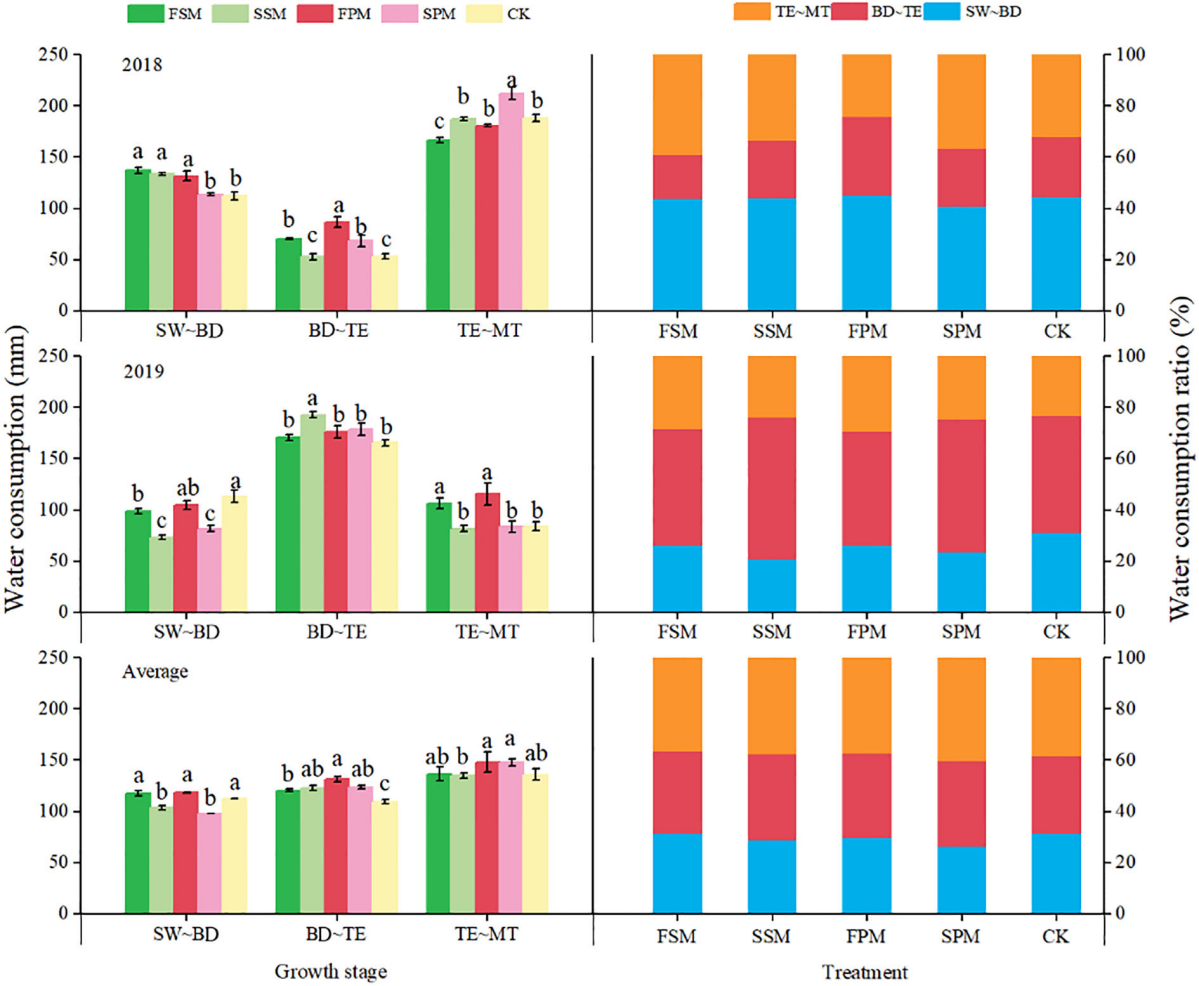
Water consumption at different growth stages of potato in 2018, 2019 and average over both growing seasons. SW~BD, during the period from planting to budding stages; BD~TE, during the period from budding to tuber expansion stages; TE~MT, during the period from tuber expansion to mature stages. FSM, maize straw strip mulching in fall; SSM, maize straw strip mulching in spring; FPM, the large ridges with black plastic film mulching and small furrows in fall; SPM, large ridges with black plastic film mulching and small furrows in spring; CK, traditional bare-land planting without mulching. Identical lowercase letters indicate no significant differences (*P > 0.05*, duncan’s multiple range test) among potato treatments in a given year. Bars represent the standard error.

The mulching treatments significantly influenced water consumption at each growing stage of potato (*P < 0.05*) (**Figure 4**). In the 2018 growing season, compared to CK, the FSM and SSM treatments significantly increased water consumption during the period from planting to budding stages by 22.3% and 19.2% (*P < 0.05*), respectively. Among the PM treatments, only FPM significantly (*P < 0.05*) increased water consumption compared to CK (17.6%). During the period from budding to tuber expansion stages, FPM and the SPM significantly (*P < 0.05*) improved water consumption by 62.9% and 32.6%, respectively, compared to CK. Among the SM treatments, only FSM significantly (*P < 0.05*) increased water consumption compared to CK (32.6%). Additionally, FSM and SPM significantly (*P < 0.05*) improved the water consumption during the period from tuber expansion to maturity stages by 11.4% and 12.7%, respectively, compared to CK.

In the 2019 growing season, compared to CK, the FSM, SSM, FPM, and SPM treatments significantly (*P < 0.05*) reduced the water consumption during the period from planting to budding stages by 12.9%, 36.3%, 7.5%, and 27.8% on average, respectively. The SSM treatment significantly (*P < 0.05*) increased water consumption during the period from budding to tuber expansion stages; compared to CK (16.4%), while no significant differences (*P > 0.05*) were observed under other treatments. During the period from tuber expansion to maturity stages, FSM and FPM significantly (*P < 0.05*) increased water consumption compared to CK, by 26.6% and 22.2%, respectively. However, no significant differences (*P > 0.05*) were observed between the spring mulching treatments and CK during these stages.

When comparing mulching stages across the two growing seasons, water consumption under FPM (11.1%) was higher than under SPM (3.1%) compared to CK. Additionally, SSM and SPM significantly (*P < 0.05*) reduced water consumption between the planting and budding stages by 8.2% and 9.8%, respectively, compared to CK. During the period from budding to tuber expansion stages, water consumption under SSM (12.3%) was higher than under FSM (3.1%), while water consumption under FPM (20.3%) was higher than that under SPM (13.1%). However, no significant differences (*P* > 0.05) were observed between the mulching treatments and CK during the period from tuber expansion to maturity stages.

### 3.3 Water consumption intensity at different potato growth stages

**Figure 5:**
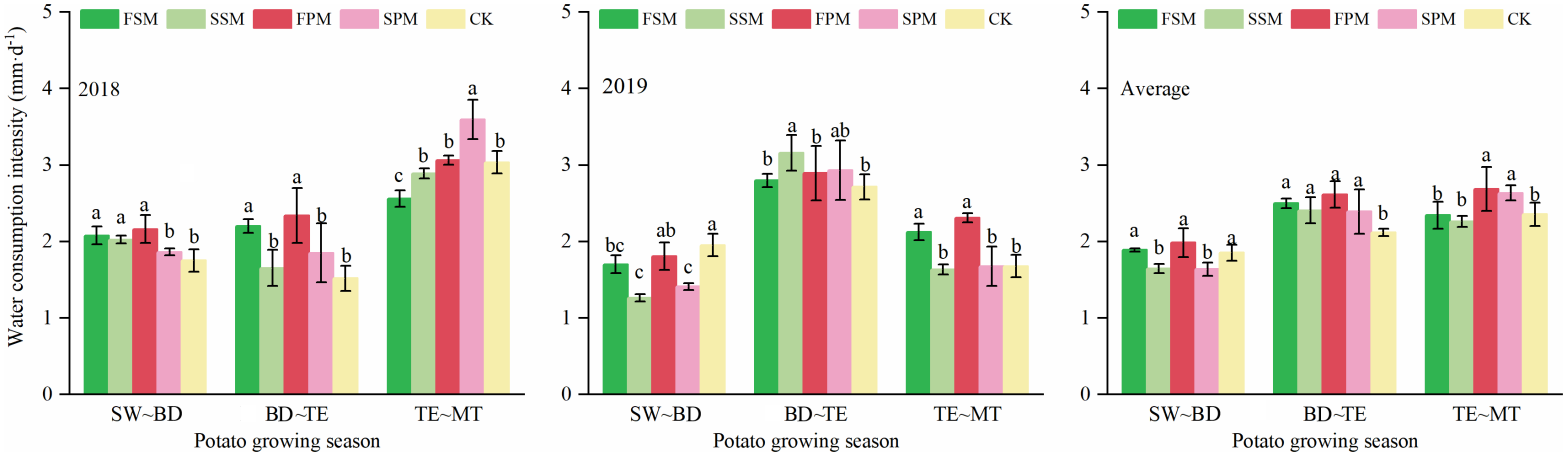
Water consumption intensity at different growth stages of potato in 2018, 2019 and averaged over both growing seasons. SW~BD, SW~BT, during the period from planting to budding stages; BD~TE, during the period from budding to tuber expansion; TE~MT, during the period from tuber expansion to mature. FSM, maize straw strip mulching in fall; SSM, maize straw strip mulching in spring; FPM, large ridges with black plastic film mulching and small furrows in fall; SPM, large ridges with black plastic film mulching and small furrows in spring; CK, traditional bare-land planting without mulching. Identical lowercase letters indicate no significant differences (*P > 0.05*, duncan's multiple range test) among potato treatments in a given year. Bars represent the standard error.

Under the mulching treatments, the WCI of potato during the entire growth stage exhibited a generally increasing trend (**Figure 5**). In the 2018 growing season, during the period from planting to budding stages, compared to CK, FSM, SSM and FPM significantly *(P < 0.05)* increased the mean WCI by 0.3, 0.3, and 0.4 mm·d^-1^, respectively. In contrast, no significant (*P > 0.05*) difference was observed between SPM and CK. During the period from budding to tuber expansion stages, FSM and FPM significantly (*P < 0.05*) increased the WCI by 0.7 mm·d^-1^ and 0.8 mm·d^-1^, respectively, compared to CK, while no significant (*P > 0.05*) differences were observed between SPM and CK. During the period from tuber expansion to maturity stages, FSM significantly (*P < 0.05*) reduced the WCI by 0.5 mm·d^-1^, compared to CK, whereas SPM significantly (*P < 0.05*) increased it by 0.6 mm·d^-1^. No significant (*P > 0.05*) differences were observed between SSM, FPM, and CK during this stage.

In the 2019 growing season, during the period from planting to budding stages, FSM, SSM and SPM significantly (*P < 0.05*) reduced the mean WCI compared to CK, by 0.3, 0.7, and 0.5 mm·d^-1^, respectively. The SSM treatment significantly (*P < 0.05*) increased the WCI during the period from budding to tuber expansion stages compared to CK (0.4 mm·d^-1^), while no significant (*P > 0.05*) differences were observed between FSM, FPM, SPM, and CK. during the period from tuber expansion to maturity stages, compared with CK, FSM and FPM significantly (*P < 0.05*) increased the WCI by 0.5 mm·d^-1^ and 0.6 mm·d^-1^, respectively, compared to CK, with no significant difference (*P > 0.05*) between the spring mulching treatments and CK.

Averaged across the two growing seasons, compared to CK, SSM and SPM significantly (*P < 0.05*) reduced the WCI between the planting and budding stages by 0.2 mm·d^-1^ and 0.2 mm·d^-1^, respectively. During the period from budding to tuber expansion stages, SM and PM significantly (*P < 0.05*) improved the mean WCI by 0.3 mm·d^-1^ and 0.4 mm·d^-1^, respectively. The increase in mean WCI for the different mulching stages compared to CK followed the order: FSM (0.4 mm·d^-1^) > SSM (0.3 mm·d^-1^) and FPM (0.50 mm·d^-1^) > SPM (0.3 mm·d^-1^). Additionally, the PM significantly (*P < 0.05*) increased the mean WCI by 0.3 mm·d^-1^ during the period from tuber expansion to maturity stages compared to CK.

SW~BD, SW~BT, during the period from planting to budding stages; BD~TE, during the period from budding to tuber expansion; TE~MT, during the period from tuber expansion to mature. FSM, maize straw strip mulching in fall; SSM, maize straw strip mulching in spring; FPM, large ridges with black plastic film mulching and small furrows in fall; SPM, large ridges with black plastic film mulching and small furrows in spring; CK, traditional bare-land planting without mulching. Identical lowercase letters indicate no significant differences (*P > 0.05*, duncan’s multiple range test) among potato treatments in a given year. Bars represent the standard error.

### 3.4 WUE and yield

Mulching treatments significantly (*P < 0.05*) increased the fresh yield, dry yield, and WUE of potato (**Table 2**). In the 2018 growing season, compared to the CK, SM and PM significantly (*P < 0.05*) increased fresh potato yield by 15.8% and 33.3%, dry potato yield by 15.7% and 32.1%, and WUE by 9.3% and 18.7%, respectively. In 2019, the SM and the PM significantly (*P < 0.05*) increased fresh potato yield by 13.7% and 18.3%, and WUE by 9.1% and 10.4%, respectively. When comparing the treatments, the increase in fresh yield followed the order: FPM (35.3%) > SPM (31.2%) > FSM (16.1%) > SSM (15.4%) in 2018, and FPM (27.1%) > FSM (19.5%) > SPM (9.6%) > SSM (8.0%) in 2019.

**Table 2:** Changes in potato yield and water use efficiency under different treatments (2018–2019).

Year	Treatment	Fresh potato yield	Dry potato Yield	weight per fresh tuber	tuber weight per plant	Commercial potato rate	Water use efficiency
		(kg·ha^-1^)	(kg·ha^-1^)	(g)	(g)	(%)	(kg·ha^-1^·mm^-1^)
2018	FSM	31878.7c	7866.4b	87.1b	759.0c	80.6b	21.0b
	SSM	31693.0c	7817.2b	87.1b	754.6c	80.4b	20.9b
	FPM	37176.5a	9161.2a	115.7a	885.2a	86.0a	23.0a
	SPM	36052.2b	8886.3a	117.6a	858.4b	87.5a	22.6a
	CK	27459.2d	6777.2c	85.0b	653.8d	73.9c	19.2c
	CV (%)	11.8	11.8	16.8	11.8	6.6	7.1
2019	FSM	40040.0b	9114.4b	103.5b	762.7b	80.5b	24.3ab
	SSM	36167.2c	8114.3c	103.4b	688.9c	80.2b	23.3b
	FPM	42569.2a	9813.2a	118.4a	810.8a	85.1a	24.7a
	SPM	36714.8c	8069.0c	116.0a	699.3c	87.1a	23.4b
	CK	33497.7d	7913.2c	93.9c	638.1d	75.5c	21.8c
	CV (%)	9.4	9.6	9.4	9.4	5.6	4.8
Average	FSM	35959.3b	8490.4b	95.3b	760.8b	80.6c	22.6b
	SSM	33930.1c	7965.7c	95.2b	721.7c	80.3c	22.1b
	FPM	39872.8a	9487.2a	117.0a	848.0a	85.6b	23.8a
	SPM	36383.5b	8477.7b	116.8a	778.9b	87.3a	23.0ab
	CK	30478.4d	7345.2d	89.5c	645.9d	74.7d	20.5c
	CV (%)	9.8	9.4	12.8	9.9	6.1	5.6

Different lower letters indicate significant differences between treatments (*P < 0.05*).

The yield improvement under mulching was primarily attributed to significant increases in tuber weight per plant and single tuber weight. In the 2018 growing season, compared to CK, FPM and SPM significantly (*P < 0.05*) increased single tuber weight by 36.2% and 38.3%, respectively. Similarly, SPM, FPM, FSM, and SSM significantly (*P < 0.05*) increased tuber weight per plant by 16.1%, 15.4%, 35.4%, and 31.3%, respectively, and improved the commodity rate by 15.4%, 12.8%, 6.7%, and 6.3%, respectively. In the 2019 growing season, compared to CK, all mulching treatments significantly (*P < 0.05*) increased single tuber weight and tuber weight per plant by 17.4% and 16.0%, respectively. Additionally, SPM, FPM, FSM, and SSM significantly (*P < 0.05*) increased the commercialization potato rate by 15.4%, 12.8%, 6.7%, and 6.3%, respectively.

Averaged across the two growing seasons, SM and PM significantly (*P < 0.05*) increased fresh potato yield by 14.7% and 25.1%, dry potato yield by 12.0% and 22.3%, and WUE by 9.2% and 14.3%, respectively. The increase in fresh potato yield, dry potato yield, and WUE under PM treatments was greater than under SM treatments. Furthermore, fall mulching outperformed spring mulching in enhancing these variables. The results indicated that the increase in single tuber weight played a crucial role in improving commercialization potato rate and overall yield of potato.

### 3.5 Correlation between stage water consumption, water consumption intensity, and potato yield

**Figure 6:**
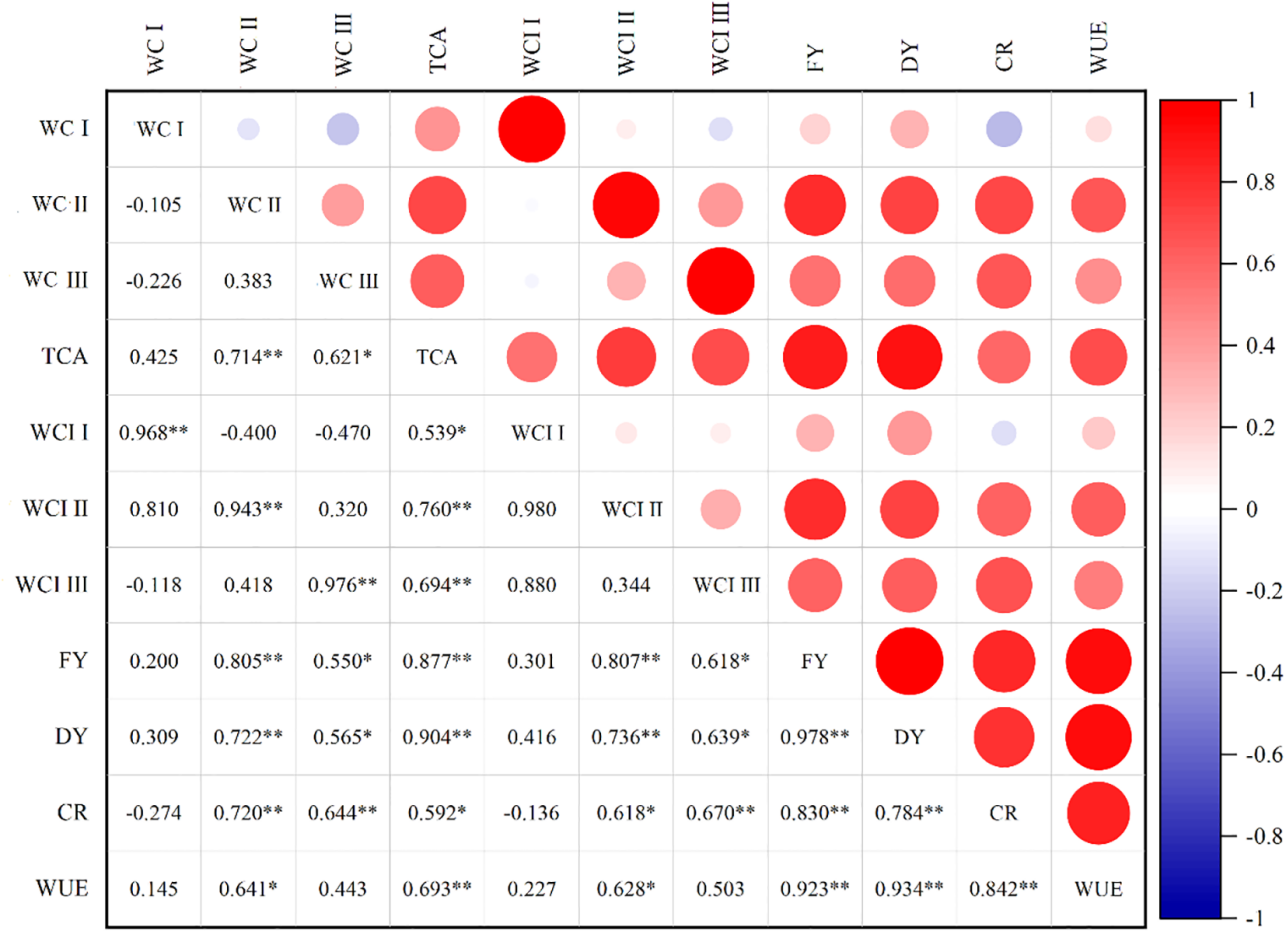
Correlation indexes between water consumption and potato yield (2018–2019) under different treatments. WC I, Soil water consumption during the period from planting to budding stages; WC II, Soil water consumption during the period from budding stages tuber expansion; WC III, Soil water consumption during the period from tuber expansion to mature stages; TCA, total water consumption amount in the growth stage; WCI I, soil water consumption intensity during the period from planting to budding; WCI II, soil water consumption intensity during the period from budding to tuber bulking; WCI III, soil water consumption intensity during the period from tuber expansion to mature stages; FY, fresh potato yield; DY, dry potato yield; CR, commercialization potato rate; WUE, water use efficiency. * and ** indicate significant correlation at *P < 0.05* and *P < 0.01*, respectively.

As illustrated in **Figure 6**, significant relationships were observed between WC, WCI, WUE, and yield. Fresh potato yield exhibited significant positive correlations with WC and WCI during the period from budding to tuber expansion stages (r = 0.805–0.807, *P < 0.01*) and during the period from the tuber expansion to maturity stages (r = 0.550–0.618, *P < 0.05*). Similarly, WUE showed significant positive correlations with the water consumption during the period from budding to tuber expansion stages (r = 0.641, *P < 0.05*) and with WCI during the period from the tuber bulking to maturity stages (r = 0.628, *P < 0.05*).

Furthermore, water consumption during the middle and late growing stages (BD-TE and TE-MT) demonstrated significant positive correlations with single tuber weight (r = 0.692–0.724, *P < 0.01*), tuber weight per plant (r = 0.564–0.808, *P < 0.01*), and the commercialization potato rate(r = 0.644–0.720, *P < 0.01*). These results indicated that the higher water consumption during these critical growth stages contributed to increased tuber yield and WUE. Additionally, mulching treatment enhanced W by effectively reducing ineffective water consumption during the middle and late stages. This optimization of water use promoted greater weight per fresh tuber, tuber weight per plant, and commodity rate, ultimately improving potato yield and quality.

## 4 Discussion

In semi-arid rainfed areas of China, soil water and precipitation are the primary sources of water for potato growth (Chang et al., 2020). Mulching practices, such as straw mulch and plastic mulch, play a crucial role in enhancing soil water retention and improving the utilization efficiency of precipitation. These practices inhibit soil evaporation, optimize the soil–water environment, and significantly promote potato growth (Dong et al., 2022; Sabet et al., 2022; Singh et al., 2015). In this study, In this study, both enhanced the soil water supply capability and increased soil water storage in rainfed agricultural areas. This improvement ensured more soil water was available for potato growth throughout the root zone. The effectiveness of mulching can be attributed to its ability to enhance precipitation infiltration, prevent soil water evaporation, and facilitate the utilization of water stored in deeper soil layers (Chen et al., 2019b; Yang et al., 2019).

Under the ridge-furrow system with full plastic film mulching, rainwater harvesting efficiency is significantly improved, soil moisture consumption is reduced, and soil water storage is substantially increased in the semi-arid areas of Northwest China (Jiang and Li, 2015; Xie et al., 2020). In this study, both SM and PM increased soil water storage in the 0–200 cm soil layer during both growing seasons. However, fall mulching demonstrated greater benefits compared to spring mulching. This is likely because the fall mulching effectively increases the precipitation utilization efficiency (the proportion of rainfall converted to plant-available soil water), reduces soil water evaporation during the fall and winter resting stages, and ensures more adequate soil water availability for potato emergence (Qin et al., 2022).

In the rainfed semi-arid regions of Northwest China, relying solely on natural rainfall often fails to meet the water demands of crops during critical growth stages. Therefore, optimizing water consumption patterns through mulching practices is essential to ensure sufficient soil moisture and mitigate the risks of extreme drought (Chai et al., 2022a; Chen et al., 2019a). Numerous studies have demonstrated that mulching significantly improves water consumption structure effectively by reducing evaporation and enhancing WUE (Hou and Li, 2019; Zhao et al., 2019b; Zhao et al., 2014). In this study, both straw mulch and plastic mulch significantly modulated the soil water consumption pattern across the two growing seasons. A key finding was that spring-applied mulches (SSM and SPM) consistently reduced soil water consumption during the early growth stage, while fall-applied mulches (FSM and FPM), which had already conserved winter precipitation, sometimes led to higher early-season soil water consumption due to more available water. Crucially, all mulching treatments tended to support higher soil water consumption during the critical middle and later stages compared to CK. Consistent with our results presented in section 3.1, the PM system generally led to a greater increase in soil water storage than the SM system. However, the improvements under SM were also significant and contributed to the enhanced yield. Fall mulching outperformed spring mulching overall. This is primarily because fall mulching effectively retains precipitation and reduces soil evaporation during the spring, leading to better soil moisture conservation compared to spring mulching (Tao et al., 2015).

Mulching practices effectively modify the crop growth environment in rainfed agriculture (Zheng et al., 2021). For instance, while ridge and furrow mulching systems reduce water consumption (WC) during the early growth stages, they increase it in the mid-stage, with no clear pattern observed later. Study has also shown that combining plastic film mulching on ridges with straw mulching in furrows significantly restrained soil evaporation, remarkably enhanced plant transpiration (Zhang et al., 2020c). These practices improve effective soil water storage by 12.4-34.1%, increase potato yields by 1.0-27.4%, and boost WUE by 12.9-207.5% compared to non-mulched treatments (Amare and Desta, 2021; Chen et al., 2019a; Qi et al., 2020; Singh et al., 2021). Similar results were observed in the present study, SM and PM increased dry potato yield by 12.0% and 22.3%, and WUE by 9.2 and 14.3%, respectively. These improvements were achieved by enhancing water consumption during the critical period from tuber bulking to starch accumulation stages (by 7.7% and 16.7% for SM and PM, respectively), which was supported by an overall increase in seasonal soil water storage. Additionally, mulching significantly reduced WCI before the budding and increased it after the tuber expansion stage, easing water demand during the late growth stage (Li et al., 2018). Similar results were observed in this study, probably because in arid and semi-arid areas, mulching helps to reduce soil surface evaporation and conserve soil moisture resulting in a lower water consumption intensity in the early stages of growth. As the crop grows, its water demand increases. However, mulch can moderate the increase in water consumption intensity during the later stages by regulating soil condition in the two growing seasons, specifically, SSM and SPM increased the WCI 0.3 mm·d^-1^ and 0.4 mm·d^-1^ during the period from budding to tuber expansion stages respectively, while decreased WCI by 0.2 mm·d^-1^ and 0.2 mm·d^-1^ during the period from planting to budding stages. The mechanism behind this yield improvement is clearly explained by our correlation analysis (**Figure 6**). We found that potato yield and WUE were most strongly and positively correlated with water consumption during the period from budding to tuber expansion stages (BD-TE) and the tuber expansion to maturity stages (TE-MT). This indicates that the primary benefit of both SM and PM systems was not just in saving water, but in shifting the water supply to these most critical growth phases, thereby promoting tuber bulking and increasing single tuber weight and commodity rate (Chen et al., 2019a), as shown in our results (Section 3.4).

Mulching cultivation is a widely adopted practice in the rainfed semi-arid regions of Northwest China, proven to enhance crop yields and water use efficiency (WUE) by improving soil moisture conservation and utilization under limited rainfall (Dong et al., 2018; Duan et al., 2021; Xiao et al., 2019). Consistent with previous findings on ridge and furrow systems (Qin et al., 2014; Liang et al., 2018), our study confirmed that both plastic film (PM) and straw strip mulching (SM) create stable moisture conditions, promoting seedling emergence, growth, and ultimately increasing tuber weight and yield. This demonstrates that mulching facilitates the effective collection and utilization of precipitation, raises soil moisture content, and ensures water supply during key reproductive periods, thereby supporting potato growth and yield enhancement. Long-term research on maize aligns with this, showing that mulching boosts yield and WUE by improving soil water retention and precipitation use efficiency (Zhang et al., 2022). Similarly, studies on potato confirm that PM and SM enhance total water consumption by reducing soil evaporation and promoting crop transpiration, ultimately improving yield and WUE in semi-arid areas (Chang et al., 2020). However, some studies have reported that SM has a greater effect than PM on increasing potato tuber yield and WUE under dry conditions, particularly when combined with fall ploughing after the autumn harvest (Hou et al., 2020). This contrasts with the findings of this study, likely because PM offers greater advantages in soil water conservation, evaporation reduction, and rainwater harvesting under conditions of relatively sufficient rainfall. Mulching periods and mulching methods together determine potato yield and WUE. The generally superior performance of the PM system over the SM system in this study can be attributed to the integrated ridge-furrow design, which more effectively harvests rainwater and the impermeable film that provides a robust physical barrier against evaporation. The increase in fresh potato yield, dry potato yield, and WUE under PM treatments was higher than that under SM treatments. It is worth noting that the effectiveness of straw strip mulching (SM) in this study may be affected by the continuous use of the same batch of straw. It has been shown that repeated use of the previous year’s straw can lead to a decrease in the mulching effect (e.g., weakened moisture retention capacity, reduced stability of the mulch layer, etc.) due to its partial decay and decomposition (Zhao et al., 2019a). This factor may partly explain why the SM treatment was less effective than the PM treatment in increasing yields. In contrast, PM does not suffer from material degradation and can maintain a more stable cover. Although PM performed better in this trial, the sustainability advantage of SM should not be overlooked. By returning straw to the field, SM can release soil nutrients, thus saving fertilizer costs (Gao et al., 2022b); at the same time, it avoids mulch contamination, and replacing mulch also saves mulch costs, which is expected to save about 214 USD per hectare. Although SM temporarily lagged behind PM in terms of yield increase in the specific data of this study, its comprehensive benefits in terms of environmental friendliness and resource recycling were significant. In addition, some studies have found that straw mulching may increase the occurrence of pests and diseases (McNee et al., 2022), which was not confirmed in this experiment, and straw strip mulching did not cause pest and disease problems in Northwest China. Therefore, the choice between these two mulching systems represents a trade-off. The PM system offered higher immediate yield benefits, whereas the SM system provides a sustainable and cost-effective alternative with significant environmental benefits. Taken together, fall straw strip mulching is expected to be an alternative to mulching with the potential to be economical, green, and yield-enhancing at the same time.

## 5 Conclusion

Both straw strip mulching and plastic film mulching can optimize the water consumption structure by reducing the water consumption during the early growth stages of potato and increasing it during the middle and later stages. Additionally, fall mulching is more advantageous in improving potato yield and water use efficiency. However, compared with plastic film mulching, straw strip mulching not only reduces production costs, but also avoids the problem of plastic film residue pollution. Straw strip mulching did not significantly increase the risk of pests, diseases and weeds in the practice of rainfed agriculture in the Northwest. Therefore, considering yield, cost and environmental sustainability, straw strip mulching is a productive, practical and environmentally friendly mulching method for potato production in this region.

## Data Availability

The raw data supporting the conclusions of this article will be made available by the authors, without undue reservation.

## Glossary

CK: traditional bare-land planting without mulching
CR: the commercialization potato rate
DY: dry potato yield
WC: Soil water consumption
FPM: large ridges and small furrows with only ridges mulched with black plastic film in fall
FSM: straw strip mulching in fall
FY: fresh potato yield
P: precipitation
PM: black plastic film mulching
PW: per plant potato weight
h: soil depth
SM: straw strip mulching
SPM: large ridges and small furrows with only ridges mulched with black plastic film in spring
SPW: single potato weight
SSM: straw strip mulching in spring
∆W: change in soil water storage
SWC: soil water content
W: soil water storage
WC: water consumption
WCI: water consumption intensity
WUE: water use efficiency
